# Gasless versus gas-inflated transaxillary endoscopic thyroidectomy for papillary thyroid carcinoma: a cohort study on surgical outcomes and learning curves

**DOI:** 10.3389/fendo.2025.1710612

**Published:** 2025-11-05

**Authors:** Qi Zhao, Bin Lv, Lei Sheng, Nan Liu

**Affiliations:** Department of Thyroid Surgery, General Surgery, Qilu Hospital of Shandong University, Jinan, Shandong, China

**Keywords:** papillary thyroid carcinoma, gasless transaxillary endoscopic thyroidectomy, gas-inflated transaxillary endoscopic thyroidectomy, conventional open thyroidectomy, cumulative sum

## Abstract

**Background:**

Conventional open thyroidectomy (COT) results in visible neck scarring. Transaxillary endoscopic thyroidectomy (TET) comprises gasless (suspension-assisted) and gas-inflated approaches, both of which offer superior scar concealment. This study aimed to compare the efficacy and safety of these two endoscopic techniques for treating papillary thyroid carcinoma (PTC).

**Methods:**

A total of 471 patients were stratified into three groups: gasless transaxillary endoscopic thyroidectomy (GTET), gas-inflated transaxillary endoscopic thyroidectomy (GITET), and COT. Comparative analyses included perioperative outcomes, complication rates, cosmetic satisfaction and others. The cumulative sum (CUSUM) curve was applied to evaluate the learning curves of GTET and GITET.

**Results:**

Patients in the TET groups were younger and included a higher proportion of females compared to COT. The COT group demonstrated advantages in operation time, postoperative drainage volume, and the number of retrieved central lymph nodes over TET groups. No significant differences were observed among the three groups in postoperative complication rates or sensory abnormalities. However, the COT group had higher swallowing-discomfort incidence. In terms of cosmetic outcomes, GITET surpassed GTET, with lower postoperative pain scores. The learning curves for both GTET and GITET were biphasic, achieving mastery after 42 and 67 cases respectively. No significant difference was found in the efficacy of central lymph node dissection between the two endoscopic approaches.

**Conclusion:**

Both GTET and GITET were reliable and safe surgical approaches, with reduced postoperative swallowing discomfort compared to COT. While GITET offered superior cosmetic outcomes and lower postoperative pain scores than GTET, achieving technical proficiency required more cases.

## 1 Introduction

For decades, the global incidence of thyroid carcinoma (TC), predominantly papillary thyroid carcinoma (PTC), has risen steadily (1). Surgical intervention remains the primary treatment for TC, but conventional open thyroidectomy (COT) leaves noticeable neck scars, compromising cosmesis. Endoscopic thyroidectomy (ET) offers clear surgical visualization with minimal visible scarring (2). Transaxillary endoscopic thyroidectomy (TET) has garnered particular attention from patients and surgeons for its ability to conceal incisions within natural axillary folds, thereby optimizing cosmetic results.

Gas-inflated transaxillary endoscopic thyroidectomy (GITET) was initially performed using CO2-insufflation to maintain the operative workspace (3). However, this approach entailed challenges including persistent intraoperative smoke accumulation (necessitating frequent lens cleaning); potential risks such as subcutaneous emphysema and hypercapnia further complicated its use. Subsequently, gasless transaxillary endoscopic thyroidectomy (GTET) was introduced, reducing both lens-cleaning requirements and gas-related complications while preserving adequate visualization (4, 5).

Different TET approaches present distinct trade-offs in surgical field exposure, technical accessibility, and protection of critical anatomical structures. Comparative studies between GTET and GITET remain limited. This study systematically evaluates surgical parameters, complication rates, and patient-reported outcomes among GTET, GITET, and COT. By delineating the strengths and limitations of each TET approach, we aim to guide technical refinements and optimize procedural selection in endoscopic thyroid surgery.

## 2 Materials and methods

### 2.1 Patients

We retrospectively evaluated patients with PTC undergoing unilateral thyroidectomy with central lymph node dissection (CLND) at Qilu Hospital of Shandong University between April 2021 and July 2022. All surgeries were performed by an experienced surgeon. Exclusion criteria comprised: (1) prior neck surgery or cervical radiation history, (2) tumor diameter >2.0 cm, (3) suspected lateral lymph node metastasis or requirement for lateral lymph node dissection (LLND), (4) evidence of locoregional invasion or distant metastasis, (5) concurrent malignancies or severe systemic comorbidities. All diagnoses were histopathologically confirmed as PTC postoperatively. Our study was conducted in accordance with the Declaration of Helsinki, approved by the Institutional Review Board of Qilu Hospital (approval number: KYLL-202506-017), and written informed consent was obtained from all participants.

### 2.2 Surgical procedures

#### 2.2.1 COT

The patient was positioned supine under general anesthesia, with neck hyperextension facilitated by shoulder pads to optimize anterior neck exposure. The surgical field-extending from chin to upper chest-was aseptically prepared and sterilely draped. A 4–6 cm transverse incision was created approximately 2 cm superior to the sternal notch along a natural skin crease. Subcutaneous tissue and platysma were divided, followed by longitudinal elevation of a subplatysmal flap to expose the strap muscles. These muscles were then divided at the midline and retracted laterally to reveal the thyroid gland. The recurrent laryngeal nerve (RLN) was identified along the tracheoesophageal groove via the tunnel approach, with nerve integrity confirmed by intraoperative neuromonitoring (IONM). Parathyroid glands were meticulously preserved with their vascular pedicles; devascularized glands were autotransplanted into the sternocleidomastoid muscle (SCM).

#### 2.2.2 GTET

**Figure 1:**
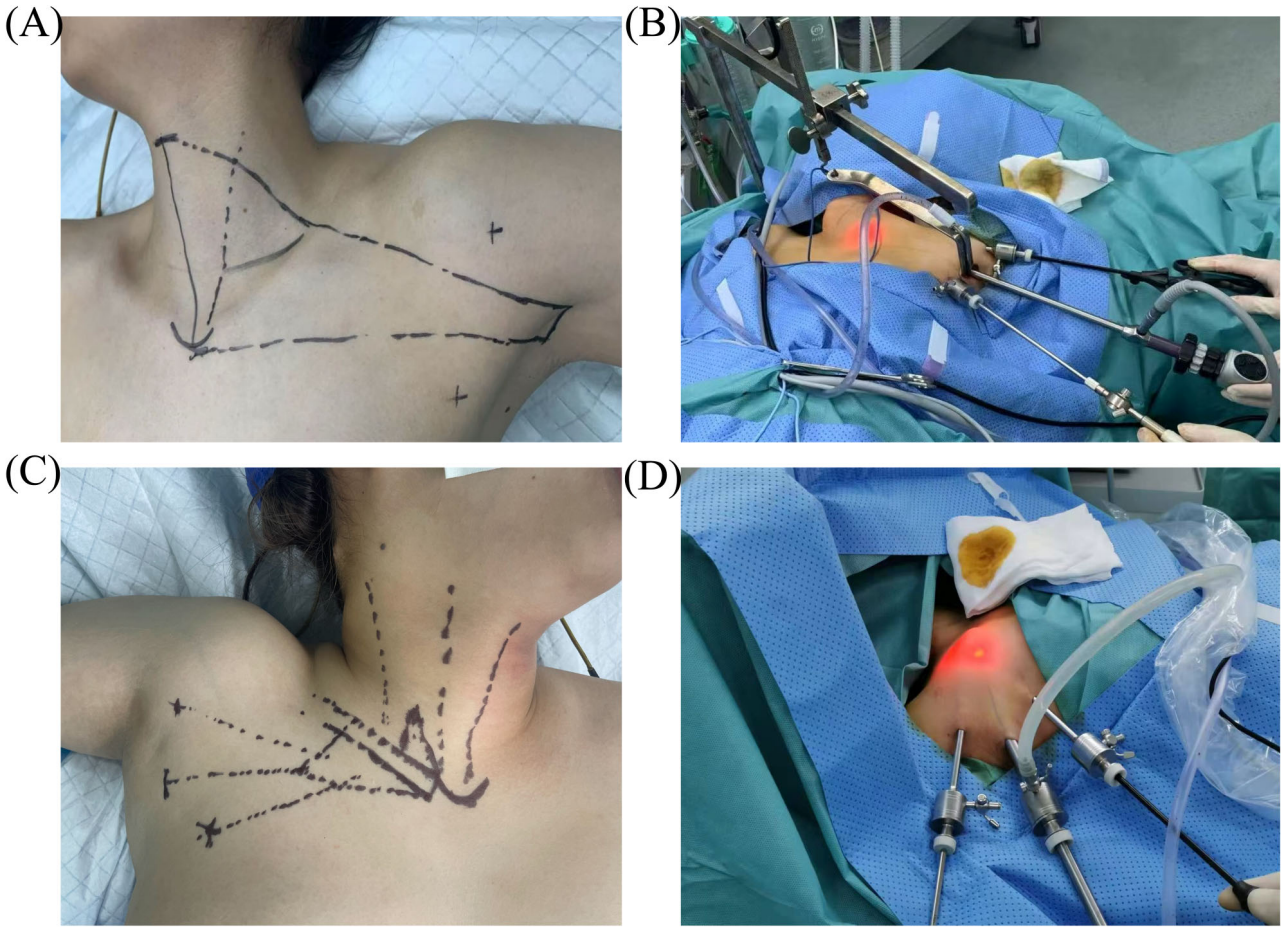
Incision and Trocar positions as well as flap dissection ranges in GTET and GITET. **(A)** Incision positions and flap dissection range in GTET. **(B)** Trocar positions in GTET. **(C)** Incision positions and flap dissection range in GITET. **(D)** Trocar positions in GITET. GTET, gasless transaxillary endoscopic thyroidectomy. GITET, gas-inflated transaxillary endoscopic thyroidectomy.

Patients were positioned identically to the COT group, with the affected arm abducted 90 degrees to optimize axillary exposure (**Figure 1A**). Following sterile preparation, a 3–5 cm longitudinal incision was created along the natural axillary crease. Subcutaneous fat and fascial layers were dissected to develop a tunnel toward the anterior neck. A custom-designed retractor, anchored to the operating table, mechanically elevated the skin and soft tissues to establish a stable gasless workspace. A 10 mm endoscope was inserted through the axillary incision, flanked by two 5 mm laparoscopic instruments arranged in a triangular configuration for optimal maneuverability (**Figure 1B**). Under endoscopic visualization, the sternal and clavicular heads of the SCM were divided using an ultrasonic scalpel. The strap muscles were dissected from the thyroid surface laterally to medially, after which the retractor was repositioned beneath the SCM sternal head and strap muscles to maintain exposure. Critical structures-including the RLN and parathyroid glands-were meticulously identified and preserved using techniques identical to the open approach.

#### 2.2.3 GITET

Patient positioning and anesthesia protocols mirrored those of GTET (**Figure 1C**). A 10 mm longitudinal incision was created along the axillary crease, followed by blunt dissection of subcutaneous tissue using a tunneling rod to establish an access pathway to the thyroid compartment. A 10 mm endoscopic trocar was inserted through the primary incision, and carbon dioxide insufflation was initiated to maintain a stable operative workspace at 6 mmHg pressure, minimizing subcutaneous emphysema risk. Two additional 5 mm incisions were created bilaterally along the axillary fold, establishing a triangular port configuration (**Figure 1D**). Under endoscopic visualization, 5 mm trocars were inserted for instrument access. The sternal and clavicular heads of the SCM were divided, and strap muscles were dissected to expose the thyroid gland using the same technique as in GTET. The sternal head of the SCM and strap muscles were retracted superiorly and secured with suspension sutures.

### 2.3 Surgical outcome assessment

We systematically analyzed patient demographics, tumor characteristics (size, multifocality, Hashimoto’s thyroiditis, and extrathyroidal extension), and perioperative parameters including operative time, central lymph node yield and metastatic node count, postoperative drainage volume, and length of hospital stay. Documented complications encompassed transient/permanent hoarseness, hypoparathyroidism, hematoma, surgical site infection, and recurrence. Drainage tubes were removed when output fell below 30 mL/day. Postoperative surveillance included thyroid ultrasonography every 3 months to monitor recurrence.

Pain intensity was assessed 24 hours postoperatively using the Faces Pain Rating Scale, categorized according to British Pain Society criteria: no pain (0), mild (1–3), moderate (4–6), or severe (7–10). Swallowing discomfort was defined as subjective dysphagia or foreign body sensation. Sensory abnormalities in the cervical/anterior thoracic regions were classified per the Medical Research Council (MRC) scale: S0 (complete loss), S1 (deep sensation only), S2 (partial superficial touch without pain perception), S3 (pain/touch with dysesthesia), S3+ (near normal), or S4 (normal) (6). Cosmetic satisfaction was graded using a 5-tier scale: quite dissatisfied, dissatisfied, average, satisfied, or quite satisfied. Standardized questionnaires administered at the 6-month follow-up evaluated swallowing function, sensory status, and cosmetic outcomes.

### 2.4 Statistical analysis

All analyses were performed using R 4.4.1. Categorical variables are reported as frequencies (percentages) and analyzed using Pearson’s chi-square or Fisher’s exact tests. Continuous variables are presented as mean ± standard deviation and median (interquartile range), with comparisons performed using Student’s t-tests or nonparametric alternatives. Statistical significance was defined as p<0.05. For multiple-group comparisons, the Bonferroni-adjusted significance threshold of p<0.0167 was applied.

The cumulative sum (CUSUM) method monitored deviations of individual surgical outcomes from target values, with the CUSUM curve representing the cumulative sum of these deviations (7); this technique is well-established for surgical learning curve assessments (8). Scatter plots and CUSUM curves were generated using Python 3.13, with operative times plotted chronologically by surgery date. The peak inflection point on the CUSUM curve served as the critical threshold, dividing cases into Phase I (learning period) and Phase II (mastery period) for subsequent comparative analysis.

## 3 Results

During the study period, 610 eligible patients underwent unilateral thyroidectomy with CLND. After propensity score matching (PSM), 471 patients were ultimately included (COT group, n=192; GTET group, n=102; GITET group, n=177), with baseline characteristics presented in **Supplementary Table S1**. The two TET groups exhibited similar mean ages (p=0.128), both of which were significantly lower than that of the COT group (p<0.001). Gender distribution was comparable between the TET groups (p=0.616), with females comprising over 90% of the patients. Among the three groups, no significant differences were observed in body mass index (BMI, p=0.065), tumor size (p=0.318), multifocality (p=0.516), Hashimoto’s thyroiditis (p=0.949), or extrathyroidal extension (p=0.358).

**Supplementary Table S2** summarized the perioperative outcomes of the three groups. No conversions to COT group occurred in either of the TET groups, and no statistically significant difference was observed in postoperative hospital stay (p=0.229). Regarding operative time, both TET groups showed comparable durations (106.80 ± 30.51 min versus 110.93 ± 22.52 min, p=0.106), respectively, both significantly longer than the COT group (61.39 ± 13.24 min, p<0.001). Postoperative drainage volume was markedly higher in TET groups versus COT (120.93 ± 39.95 ml and 137.02 ± 46.14 ml versus 84.26 ± 38.94 ml, respectively, p<0.001). Although the COT group retrieved significantly more central lymph nodes than TET groups (5.51 ± 3.93 versus 3.02 ± 2.40 and 2.50 ± 2.21, respectively, p<0.001), no significant difference was found in the number of metastatic lymph nodes between groups (p=0.117). Additionally, patients undergoing GTET reported significantly greater postoperative pain scores compared to both the GITET and COT groups (p<0.001).

The incidence of complications, including transient hoarseness (p=0.558), transient hypoparathyroidism (p=0.315), hematoma (p=0.350), and infection (p=0.592), showed no statistically significant differences. None of the groups reported permanent hoarseness or hypoparathyroidism. During the one-year follow-up, recurrence rates were comparable across all three groups (p=0.545) (**Supplementary Table S3**). One patient in the GITET group was found to have superior mediastinal lymph node metastasis within one year after surgery and subsequently underwent open surgery for dissection of the superior mediastinal lymph nodes. Notably, patients in the COT group exhibited significantly greater swallowing discomfort compared to both TET groups (p<0.001). Sensory abnormalities in the neck or anterior chest were not significantly different (p=0.136), with over half of patients reporting no notable sensory disturbances (**Supplementary Table S3**). As clearly demonstrated in **Supplementary Table S4**, cosmetic satisfaction was markedly higher in the ET groups than in the COT group, particularly among patients who underwent GITET (p<0.001).

**Figure 2:**
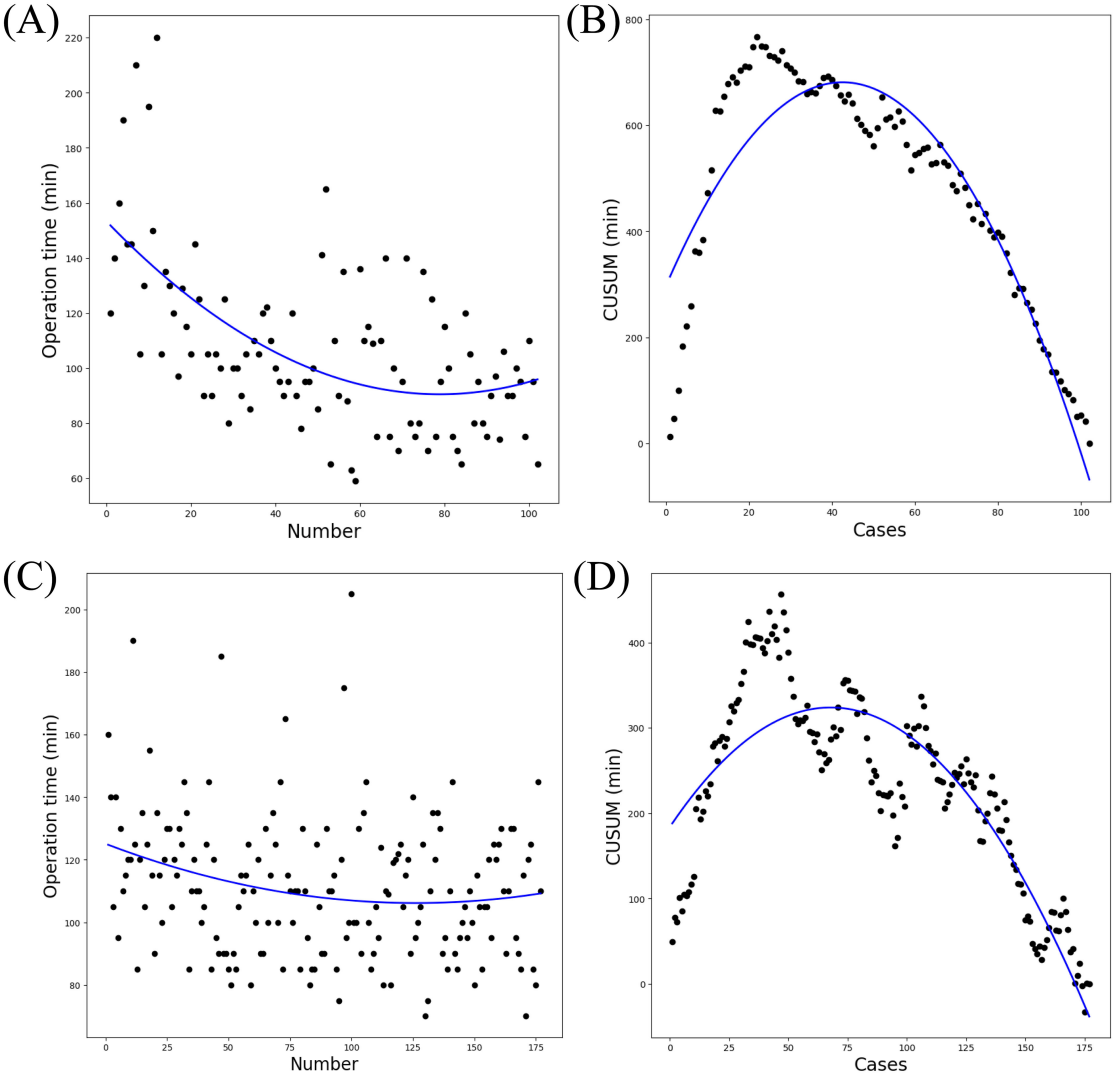
The CUSUM learning curve. Scatter plot of operative time plotted chronologically according to the sequence of performed transaxillary endoscopic thyroidectomy. **(A)** GTET. **(C)** GITET. **(B)** The CUSUM curve for GTET, divided into the learning phase (cases 1-42) and mastery phase (cases 43-102) based on the peak-corresponding case number. **(D)** The CUSUM curve for GITET, divided into the learning phase (cases 1-67) and mastery phase (cases 68-177). CUSUM, cumulative sum. GTET, gasless transaxillary endoscopic thyroidectomy. GITET, gas-inflated transaxillary endoscopic thyroidectomy.

**Figure 3:**
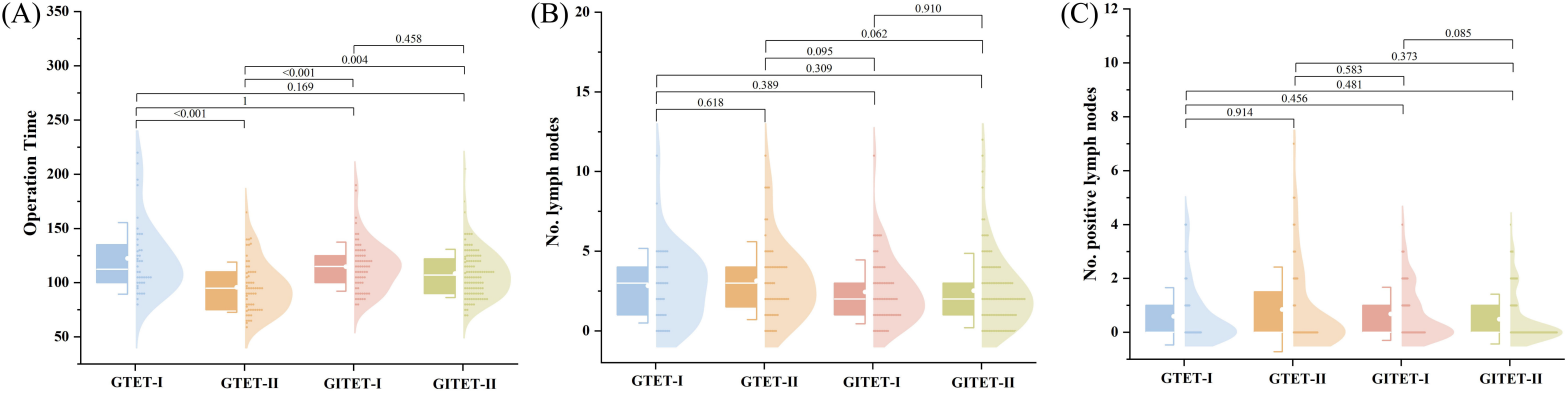
Based on the critical values, GTET and GITET were divided into GTET (learning phase), GTET-II (mastery phase), GITET-I (learning phase), and GITET-II (mastery phase), respectively, to further compare surgical outcomes across the four groups. **(A)** Operative time. **(B)** Number of central lymph nodes retrieved. **(C)** Number of positive central lymph nodes. GTET, gasless transaxillary endoscopic thyroidectomy. GITET, gas-inflated transaxillary endoscopic thyroidectomy.

The scatter plots of operative time revealed a gradual reduction in surgical duration with increasing case numbers for both the GTET and GITET groups (**Figures 2A, C**). CUSUM analysis identified inflection points at 42 cases for GTET (**Figure 2B**) and 67 cases for GITET (**Figure 2D**), enabling the division of cases into GTET-I (cases 1-42), GTET-II (cases 43-102), GITET-I (cases 1-67), and GITET-II (cases 68-177). GTET-II demonstrated significantly shorter operative times compared to GTET-I (p<0.001), while no statistically significant difference was observed between GITET-II and GITET-I (p=0.458) (**Figure 3A**). Notably, GTET-II had significantly shorter operative times than GITET-II (p=0.004). No significant differences were found in the number of retrieved central lymph nodes or metastatic lymph nodes among the four subgroups (**Figures 3B, C**).

## 4 Discussion

TC has become a significant global public health challenge, projected to emerge as China’s most prevalent malignancy by 2028 (9). The rising incidence primarily stems from increased detection of PTC (10), for which surgical resection remains the cornerstone treatment (11). This approach demonstrates superior oncological outcomes compared to ablation techniques or active surveillance (12). Given the favorable prognosis associated with early-stage TC (13), optimizing patient-centered outcomes-particularly quality of life-has become paramount in therapeutic decision-making.

COT requires a transverse incision above the sternal notch. Traction on skin flaps for surgical exposure may compromise cutaneous blood flow, potentially exacerbating scar formation (14). Moreover, scar hyperplasia and subcutaneous adhesions often contribute to swallowing discomfort, both of which are key factors diminishing quality of life (15, 16). These limitations have driven demand for endoscopic thyroidectomy, where remote-access incisions avoid visible neck scars, aligning with patient-centered cosmetic and functional priorities (17).

TET conceals incisions within the axilla when the arm is lowered, making it particularly appealing to young female patients-a preference consistently observed across studies (5, 18, 19). GTET requires an approximately 5 cm incision and creates an extensive flap from the axilla to the neck, maintained by retractor traction, whereas GITET utilizes three 1- or 0.5-cm trocars with smaller dissection areas. Our findings revealed significantly greater postoperative pain in GTET patients compared to other groups (p<0.001), likely due to the wider tissue dissection and prolonged traction time, corroborating previous reports by Liu (20).

The transaxillary approach’s remote distance from the thyroid necessitates additional time to establish and maintain the operative workspace, whether using gasless or gas-inflated techniques, resulting in significantly longer operative times compared to COT (p<0.001); prior studies have consistently supported this finding (5, 19–22). The expanded surgical dissection in both endoscopic groups led to greater drainage volumes than COT (p<0.001), though no significant difference existed between GTET and GITET (p=0.018). Notably, this increased drainage did not prolong hospitalization (p=0.229), aligning with existing literature (21, 22).

A critical challenge in endoscopic thyroidectomy concerns the debated feasibility of lymph node dissection (18). The transaxillary approach-anatomically constrained by the clavicle and sternum-resulted in fewer central lymph nodes retrieved in both GTET and GITET groups versus COT (p<0.001). However, all three groups demonstrated comparable metastatic lymph node clearance efficacy (p=0.117), consistent with Kim’s findings (5). Furthermore, previous studies suggest that for patients with clinical nodal-negative (cN0) PTC, performing prophylactic central lymph node dissection does not significantly impact surgical or oncological outcomes (23). Combined with the generally lower risk of lymph node metastasis in patients selected for endoscopic surgery, this approach sufficiently fulfills oncological safety standards. Moreover, in the lateral transaxillary approach, after complete thyroid mobilization, traction on the thyroid lower pole enables full exposure of the central lymph node compartment. When combined with a 30°endoscope, this configuration facilitates comprehensive dissection of deep central lymph nodes (18).

Safety remains paramount when evaluating surgical techniques, particularly regarding the preservation of the parathyroid gland and RLN. The literature reported transient hypoparathyroidism rated ranging from 3.15% to 64.25%, while permanent hypoparathyroidism ranged from 0% to 6.84% (24, 25). Transient RLN injury occurred in 1% to 8% of cases, with permanent RLN injury observed in 0.3% to 3.0% (26). Our study found no significant differences in complication rates among the groups, with no permanent complications observed. Transient hoarseness or hypoparathyroidism occurred in only 5% of cases, demonstrating that endoscopic approaches are non-inferior to open surgery for preserving structures. Because the transaxillary endoscopic approach follows an outward-to-inward dissection path, surgeons can more readily identify and protect the RLN and parathyroid glands. The magnified view also allows for precise distinction between lymph nodes and parathyroid tissue.

The findings of this study demonstrated that TET significantly improved patients’ quality of life. Although there was no statistically significant difference in sensory abnormalities among the three groups, the TET groups exhibited a markedly lower incidence of swallowing discomfort compared to COT, consistent with prior research (16). This phenomenon arises because COT requires transection of the platysma and creation of a subplatysmal flap to establish the operative space. The linea alba cervicalis is incised to access the thyroid gland. Postoperatively, adhesions frequently develop between this flap and the strap muscles, while connective tissue fibrosis promotes scar formation that restricts strap muscle mobility and causes swallowing discomfort. Conversely, TET establishes the operative space through dissection posterior to the strap muscles while preserving the linea alba cervicalis. This approach effectively prevents strap muscle adhesions and consequently reduces swallowing discomfort incidence (27). Regarding cosmetic outcomes, the transaxillary approach unsurprisingly yielded superior satisfaction scores. As Arora et al. documented, patients undergoing TET reported higher scar satisfaction than COT patients from two weeks post-operation to several years (28). In our study, GITET patients reported the highest satisfaction levels, as its minimal incision best aligned with their original motivation for choosing endoscopic surgery.

This study employed the CUSUM method, a controlled chart analysis that calculates the cumulative sum of deviations between observed and target values to objectively assess learning curves (29). Our analysis determined that 42 and 67 cases were required to optimize operative times for GTET and GITET, respectively, reflecting the greater technical complexity involved in establishing the working space with GITET. These findings align with existing literature on TET techniques. Kandil et al. reported a reduction in operative time after 69 transaxillary cases (30), while Sun et al. identified a 42-case learning curve for GTET, primarily dependent on workspace familiarity (29). Comparative analysis revealed that GTET demonstrated significantly reduced operative times post-proficiency (p<0.001) and outperformed GITET even during the mastery phase (p=0.004). This may be attributed to the relatively straightforward subcutaneous tunnel creation in GTET, where initial space dissection does not require endoscopic visualization, substantially reducing operative time upon achieving proficiency. Additionally, the retractor used in GTET features an integrated suction tube, which reduces smoke accumulation and decreases the frequency of lens cleaning. Consequently, the GTET group demonstrated significantly shorter operative times during the proficiency phase compared to the GITET group (31, 32). Notably, GITET showed no significant improvement in operative time after the learning curve (p=0.458). Subcutaneous tunnel creation in the GITET group is technically more demanding, as it must be performed entirely under precise endoscopic guidance and requires carbon dioxide insufflation to maintain a confined operative space. These inherent technical constraints preclude substantial reductions in operative time even after reaching the proficiency phase. Furthermore, this outcome may also reflect the surgeon’s preceding experience with the gas-inflated breast-areola approach in endoscopic thyroidectomy. Importantly, both techniques achieved satisfactory central lymph node dissection outcomes even during the learning phase.

This research has several inherent limitations. First, as a retrospective study, it is inevitably subject to selection bias, as evidenced by the demographic disparities between the TET and COT groups, which may confound outcome assessments. Second, the follow-up duration was relatively short. Finally, the modest sample size may limit the generalizability of the findings across diverse demographic populations. Future multicenter studies with larger cohorts are needed to comprehensively evaluate these surgical approaches and optimize patient management strategies.

## 5 Conclusion

TET demonstrated comparable surgical efficacy and structure preservation to COT, while significantly reducing postoperative swallowing discomfort and achieving superior cosmetic outcomes. Although GITET offered advantages over GTET in terms of reduced postoperative pain and enhanced cosmesis, it required a larger case volume to attain technical proficiency. Future development should focus on optimizing surgical access and improving instrument design to further advance TET techniques.

## Data Availability

The raw data supporting the conclusions of this article will be made available by the authors, without undue reservation.
