# Supplementary material for: Gasless versus gas-inflated transaxillary endoscopic thyroidectomy for papillary thyroid carcinoma: a cohort study on surgical outcomes and learning curves
Source: Front Endocrinol (Lausanne). 2025 Nov 5;16:1710612. doi: 10.3389/fendo.2025.1710612 (PMC12626860; doi:10.3389/fendo.2025.1710612)
Supplement: Supplementary file 1 [file Table1.docx]

Supplementary Material

# Supplementary Tables

Supplementary Table 1. Baseline clinical characteristics of included patients after propensity score matching.

|  | **GTET (1)**  **n=102** | **GITET (2)**  **n=177** | **COT (3)**  **n=192** | ***P* value** | **(1) versus (2)** | **(1) versus (3)** | **(2) versus (3)** |
| --- | --- | --- | --- | --- | --- | --- | --- |
| **Age** | 35.62±7.13  35(30-40) | 33.99±7.01  34(30-38) | 40.08±9.90  39(33-47.75) | <0.001 | 0.128 | <0.001 | <0.001 |
| **Gender (Male/Female)** | 6/96 | 8/169 | 32/160 | <0.001 | 0.616 | 0.009 | <0.001 |
| **BMI** | 23.59±3.07  23.48(21.58-25.65) | 23.24±3.05  22.76(21.02-25.33) | 23.66±2.59  23.48(21.91-25.78) | 0.065 | 0.164 | 0.846 | 0.020 |
| **Tumor Size** | 0.68±0.35  0.60(0.40-0.93) | 0.65±0.32  0.60(0.40-0.80) | 0.67±0.29  0.60(0.50-0.80) | 0.318 | 0.482 | 0.694 | 0.118 |
| **Multifocality** | 15 (14.71%) | 22 (12.43%) | 32 (16.67%) | 0.516 | 0.589 | 0.662 | 0.250 |
| **Hashimoto Thyroiditis** | 23 (22.55%) | 39 (22.03%) | 45 (23.44%) | 0.949 | 0.921 | 0.863 | 0.748 |
| **Extrathyroidal Extension** | 64 (62.75%) | 103 (58.19%) | 104 (54.17%) | 0.358 | 0.455 | 0.157 | 0.436 |

GTET: gasless transaxillary endoscopic thyroidectomy. GITET: gas-inflated transaxillary endoscopic thyroidectomy. COT: conventional open thyroidectomy.

Supplementary Table 2. Intraoperative and postoperative conditions.

|  | **GTET (1)**  **n=102** | **GITET (2)**  **n=177** | **COT (3)**  **n=192** | ***P* value** | **(1) versus (2)** | **(1) versus (3)** | **(2) versus (3)** |
| --- | --- | --- | --- | --- | --- | --- | --- |
| **Conversions to open surgery** | 0 | 0 | 0 | - | - | - | - |
| **Operation time (min)** | 106.80±30.51  100.0(89.5-120.5) | 110.93±22.52  110(95-125) | 61.39±13.24  60(50-70) | <0.001 | 0.106 | <0.001 | <0.001 |
| **Postoperative drainage volume (ml)** | 120.93±39.95  115.00(92.25-142.75) | 137.02±46.14  130(108-161) | 84.26±38.94  77.00(61.75-95.00) | <0.001 | 0.018 | <0.001 | <0.001 |
| **Hospital durations (d)** | 4.01±0.91  4(3-5) | 4.18±0.87  4(4-5) | 4.11±0.82  4(3-5) | 0.229 | 0.090 | 0.249 | 0.471 |
| **Number of retrieved lymph nodes** | 3.02±2.40  3(1-4) | 2.50±2.21  2(1-3) | 5.51±3.93  5(3-7) | <0.001 | 0.065 | <0.001 | <0.001 |
| **Number of positive lymph nodes** | 0.75±1.38  0(0-1) | 0.56±0.95  0(0-1) | 0.97±1.71  0(0-1) | 0.117 | 0.779 | 0.183 | 0.049 |
| **Pain score** |  |  |  | <0.001 | <0.001 | <0.001 | 0.020 |
| **No pain (0)** | 0 | 0 | 0 |  |  |  |  |
| **Mild pain (1-3)** | 71 (69.61%) | 169 (95.48%) | 175 (91.15%) |  |  |  |  |
| **Moderate pain (4-6)** | 31 (30.39%) | 8 (4.52%) | 17 (8.85%) |  |  |  |  |
| **Severe pain (7-10)** | 0 | 0 | 0 |  |  |  |  |

GTET: gasless transaxillary endoscopic thyroidectomy. GITET: gas-inflated transaxillary endoscopic thyroidectomy. COT: conventional open thyroidectomy.

Supplementary Table 3. Analysis of postoperative complications.

|  | **GTET (1)**  **n=102** | **GITET (2)**  **n=177** | **COT (3)**  **n=192** | ***P* value** | **(1) versus (2)** | **(1) versus (3)** | **(2) versus (3)** |
| --- | --- | --- | --- | --- | --- | --- | --- |
| **Temporary hoarseness** | 3 (2.94%) | 10 (5.65%) | 7 (3.65%) | 0.558 | 0.386 | 1 | 0.359 |
| **Permanent hoarseness** | 0 | 0 | 0 | - | - | - | - |
| **Temporary hypoparathyroidism** | 5 (4.90%) | 4 (2.26%) | 4 (2.08%) | 0.315 | 0.295 | 0.284 | 1 |
| **Permanent hypoparathyroidism** | 0 | 0 | 0 | - | - | - | - |
| **Hematoma** | 1 (0.98%) | 1 (0.56%) | 0 | 0.350 | 1 | 0.347 | 0.480 |
| **Infection** | 0 | 1 (0.56%) | 0 | 0.592 | 1 | - | 0.480 |
| **Recurrence** | 0 | 1 (0.56%) | 3 (1.56%) | 0.545 | 1 | 0.554 | 0.624 |
| **Discomfort in swallowing** | 3 (2.94%) | 7 (3.95%) | 33 (17.19%) | <0.001 | 0.751 | <0.001 | <0.001 |
| **Abnormal sensation around the incision** |  |  |  | 0.136 | 0.316 | 0.052 | 0.258 |
| **S0/S1** | 0 | 0 | 0 |  |  |  |  |
| **S2** | 2 (1.96%) | 4 (2.26%) | 6 (3.12%) |  |  |  |  |
| **S3** | 19 (18.63%) | 42 (23.73%) | 54 (28.13%) |  |  |  |  |
| **S3+/S4** | 81 (79.41%) | 131 (74.01%) | 132 (68.75%) |  |  |  |  |

GTET: gasless transaxillary endoscopic thyroidectomy. GITET: gas-inflated transaxillary endoscopic thyroidectomy. COT: conventional open thyroidectomy.

Supplementary Table 4. Satisfaction of the postoperative cosmetic appearance.

|  | **GTET (1)**  **n=102** | **GITET (2)**  **n=177** | **COT (3)**  **n=192** | ***P* value** | **(1) versus (2)** | **(1) versus (3)** | **(2) versus (3)** |
| --- | --- | --- | --- | --- | --- | --- | --- |
| **Cosmetic satisfaction level** |  |  |  | <0.001 | 0.001 | <0.001 | <0.001 |
| **Quite dissatisfied** | 0 | 0 | 0 |  |  |  | |
| **Dissatisfied** | 1 (0.98%) | 2 (1.13%) | 3 (1.56%) |  |  |  | |
| **Average** | 3 (2.94%) | 2 (1.13%) | 30 (15.63%) |  |  |  | |
| **Satisfied** | 48 (47.06%) | 47 (26.55%) | 101 (52.60%) |  |  |  | |
| **Quite Satisfied** | 50 (49.02%) | 126 (71.19%) | 58 (30.21%) |  |  |  | |

GTET: gasless transaxillary endoscopic thyroidectomy. GITET: gas-inflated transaxillary endoscopic thyroidectomy. COT: conventional open thyroidectomy.
